# Comparing the Pre-writing Skills of Diplegic Cerebral Palsy Children to Those of Normal Children

**DOI:** 10.7759/cureus.61352

**Published:** 2024-05-30

**Authors:** Nirvi Sharma

**Affiliations:** 1 Pediatrics, New York City Department of Education, Jersey City, USA

**Keywords:** pencil grasp, early treatment response, cerebral palsy (cp), hand-writing, pre-writing

## Abstract

Introduction: The pencil grasp and drawing patterns are specific to different age levels. So, if one knows a certain pattern for that particular age, it will guide the intervention plan for children with cerebral palsy (CP). The chances of improvement in diplegic CP are possible with the help of early intervention; therefore, early intervention is only possible if one knows the areas of delay and the age at which the intervention should be started.

Material and methods: It was a cross-sectional, case-control study. A total of 60 children were selected for the study, of which 30 (50%) were normal and 30 (50%) had diplegic cerebral palsy. A convenient sampling method is used for evaluation.

Results: The t-value for pencil grasp between the two groups, i.e., normal and CP diplegic, was 3.515 (P=0.001), revealing a significant difference in the grasp pattern of the two groups. Similarly, the t-value for drawing patterns between the two groups, i.e., normal and CP diplegic, was 5.796 (P = 0.001). A significant difference was found in the drawing patterns of both groups.

Conclusion: Our study found that diplegic CP children performed lower on the Erhardt Developmental Prehension Assessment (EDPA) and showed larger variation in the pencil grasp and drawing than the normal children.

## Introduction

Prewriting skills are the basis of beautiful handwriting, while handwriting portrays the personality of an individual. This research work is a small attempt to assess the developmental age (with the help of pencil grasp and drawing pattern) by comparing pencil grasp and drawing pattern among the normal and diplegic cerebral palsies (CP). The evaluation can be used to start an early intervention. The skills required to learn to write are called prewriting skills. These are mainly the sensorimotor skills that contribute to a child’s pre-writing skills, i.e., holding and using a pencil, as well as the ability to draw, copy, and color [1].

Many children begin to scribble on paper as early as they can grasp a writing tool. As children mature, their scribbling evolves into handwriting skills specific to their culture [2]. As compiled in public interest literature by the Kid Sense Child Development Centre, the different conditions that may make pre-writing skills difficult are: inappropriate ways of holding a pencil; not being able to control a pencil; difficulty in using isolated hand and finger movements for using objects; poor endurance for pencil-based activities; difficulty in staying within the lines when coloring; inappropriate pressure on the paper for pencil-based activities; poor upper limb strength; difficulty in coordinating both hands for two-handed tasks; and poor hand-eye coordination [3].

A study examined the sequential stages of letter acquisition in 110 children between the ages of three and five. Children were observed copying numbers, letters, a few words, and a sentence three times over four months. They found the following sequential stages of prewriting and handwriting: controlled scribbles, discrete lines, dots, or symbols; straight lines or circular upper-case letters; upper-case letters; lower-case letters; numerals; and words [2].

In spastic diplegic CP, arms are less affected than legs; however, they have uncoordinated movements in the upper extremity. The development of hand function depends not only on the motor control of the shoulder girdle, arms, and hands but also on visual, perceptual, perceptual-motor, and cognitive development. The main motor aspects of hand function involve the type of grasp, the pattern of reach, the pattern of reach as well as grasp and the pattern of release. In diplegic CP, hand function is affected because of limited joint motion, weakness, and problems with isolated finger and thumb movements [4]. So, to assist with prewriting skills, we have to check hand functions. Individuals with spastic diplegia walk with a distinct "scissors" gait. The legs cross over and move stiffly back and forth, like the blades of a pair of scissors. The muscles in the feet are stiff, causing the person's toes to point towards the sky. People with spastic diplegia generally have normal intelligence, and even their language skills are usually normal as well [5].

Operational definitions

From 1987 to 1990, the American and European CP investigators met and developed a common definition: ‘‘CP is an umbrella term covering a group of non-progressive, but often changing, motor impairment syndromes secondary to lesions or anomalies of the brain arising in the early stages of development" [6].

The definitions of CP have four core components: (1) difficulty in movement and posture; (2) it results from an unusual activity in the brain; (3) it occurs early in life; and (4) the condition is static at the time of recognition [7].

## Materials and methods

It was a cross-sectional, case-control study. The present study was approved by the institutional review board (JOTC/20130410/126). A total of 60 children were selected for the study, of which 30 (50%) were normal and 30 (50%) had diplegic cerebral palsy (without any upper limb injury). A convenient sampling method is used for evaluation. Children aged between four and six years of age (both males and females) of normal development and children having diplegic cerebral palsy in the age range of 5-12 years were included in the study. Formal, informed written consent was obtained from parents and organizations included in the study. Children with diplegic cerebral palsy without any orthopedic disease in hand, without any occupational therapy intervention, with good sitting posture, with good cognitive and visual problems, and with muscle tone ranging from 1 to 1+ were included in the study.

Intellectually impaired children (moderate to severe), any upper extremity injury in which grasp is affected, severe quadriplegic, spastic, athetoid, ataxic, and dystonic CP, and children with visual impairment were excluded from the study. The children were evaluated for muscle tone, posture, and cognitive issues. The muscle tone was assessed according to the Modified Ashworth Scale, and memory and orientation were judged by simple questions about the child’s daily routine. The posture was checked by the position and balance while sitting, and the attention was evaluated through a letter cancellation test. The children with specks were included in the study.

The child was made comfortable and relaxed. Then, the child was asked to sit on a table and chair (according to the child’s need, i.e., whether the child needed a special chair or a normal table chair). The environment was quiet and free from distraction. The posture of the child was erect, with hands on the table and feet on the floor. The lighting in the room was sufficient to avoid visual clutter; extra bright light was avoided. The test was performed on an individual child to avoid distraction from the group. The whole procedure was explained to the child in a suitable language. The instructions were clear, short, and understandable. The children were divided into two groups: group 1 (normal group) and group 2 (diplegic CP). Each individual was provided a pencil (7 mm in diameter), a crayon (1½ cm in diameter), and white paper for drawing. Pencil grasp was assessed by instructing them to hold the pencil in an attempt to draw a pattern. To assess the drawing skills, the child was given a crayon and asked to draw the pattern. The raw data were analyzed by SPSS software (version 19.0; IBM Corp., Armonk, NY, USA) to obtain the mean value, SD, and T value. Further, a t-test was applied, using the same software for significance value (p-value).

## Results

After evaluation, the scoring was done with the help of a pattern component. The pattern components were related to developmental levels, according to the Erhardt Developmental Prehension Assessment (EDPA) manual. Once the developmental pattern was found, it was plotted on the scoring graph. The sheets were according to the format of the EDPA scale. EDPA is a criterion-referenced assessment scale to evaluate prehension patterns (in both the right and left arm) to guide treatment planning [8]. The data were analyzed using SPSS version 19.0. The study included sixty children, of whom 30 (50%) were normal and 30 (50%) had diplegic CP. Among the 30 normal children, 12 (40%) were male, 18 (60%) were female, 25 (83%) were right-handed, and 5 (17%) were left-handed. Out of 30 diplegic CP children, 17 (56%) were male, 13 (44%) were female, 28 (93%) were right-handed, and 2 (7%) were left-handed (Table 1).

**Table 1:** Group characteristics (M:F ratio and hand dominance in normal and diplegic CP) N: number, M: male, F: female

	N	M	F	Right-handed	Left-handed
Normal	30	12 (40%)	18 (60%)	25 (83%)	05 (17%)
Diplegic CP	30	17 (56%)	13 (44%)	28 (93%)	02 (07%)

The t-value for chronological age was 10.610, and the p-value was 0.001, which reveals a significant difference in the chronological age of both groups. The t-value for pencil grasp between the two groups, i.e., normal and CP diplegic, was 3.515 (P=0.001), revealing a significant difference in the grasp pattern of the two groups (Table 2).

**Table 2:** t-test analysis between normal and diplegic CP N: number, SD: standard deviation, 1 = normal children; 2 = diplegic CP children *Significant difference in chronological age and pencil grasp

Group (normal vs CP)		N	Mean	SD	t-value	p-value
Chronological age	1	30	5.33	0.606	10.610	0.001*
	2	30	9.50	2.064		
Pencil grasp	1	30	5.70	0.596	3.515	0.001*
	2	30	4.85	1.183		

The t-value for chronological age was 7.503 and the p-value was 0.001, which reveals the significant difference in the age of male children taken for the test. The pencil grasp pattern in males of both groups shows a t-value of 2.348 (P-value = 0.026), which reveals that the results were significant. Similarly, in the drawing pattern, the t-value was 3.037 (P-value = 0.005), showing a significant difference (Table 3).

**Table 3:** t-test analysis between normal and diplegic CP male N: number, SD: standard deviation; 1 = normal children, 2 = diplegic CP children *Significant difference in chronological age, pencil grasp and drawing

Normal and diplegic CP male		N	Mean	SD	t-value	p-value
Chronological age	1	12	5.36	0.633	7.503	0.001*
	2	17	9.18	1.811		
Pencil grasp	1	12	5.731	0.6651	2.348	0.026*
	2	17	4.794	1.3117		
Drawing	1	12	4.929	0.6753	3.037	0.005*
	2	17	4.088	0.8336		

The chronological t-value was 7.543 (p-value = 0.001), indicating a significant age difference between the two groups. In pencil grasp performance, the t-value was 2.483 (P-value = 0.020), which shows the result is significant, i.e., there is a difference in the performance of the grasp pattern, whereas, in the drawing, the t-value was found to be 5.181 (P-value 0.001), which shows that there is a significant difference (Table 4).

**Table 4:** t-test analysis between normal and diplegic CP female N: number, SD: standard deviation, 1 = normal children, 2 = diplegic CP children *Significant difference in chronological age, pencil grasp and drawing

Normal and diplegic CP female		N	Mean	SD	t-value	p-value
Chronological age	1	18	5.31	0.602	7.543	0.001*
	2	13	9.92	2.362		
Pencil grasp	1	18	5.69	0.602	2.483	0.020*
	2	13	4.92	1.038		
Drawing	1	18	5.125	0.5916	5.181	0.001*
	2	13	3.808	0.7783		

## Discussion

Cerebral palsy is a condition in which both motor and sensory skills are affected, which affects pre-writing and handwriting skills. So, to improve the quality of life and functional outcome in cerebral palsy, it is vital to identify these areas as early as possible and start early intervention therapies to enhance the writing and pre-writing skills in cerebral palsy [9-12]. As mentioned in our study, a difference was found in pencil grasp patterns in normal and diplegic cerebral palsy children (p-value = 0.001). Also, a difference was found in chronological age and the development of prewriting skills secondary to a delay in the development of sensory and motor skills as well as a lack of early interventions.

A significant difference was found when the pencil grasp pattern emerging in the normal children was compared with the diplegic CP children (p-value 0.001). These are mainly the sensorimotor skills that contribute to a child’s prewriting skills [1]. The difference in the chronological age (t-value = 10.610; p-value = 0.001) and development of prewriting skills are because of the neurological and physiological development of CP children. Sensory information is important for hand skills as it tells the brain about feelings, movement, and the position of the hand while performing any task. It has been found that accurate sensory information is crucial for children when developing pre-writing skills. Research indicates that poor kinesthetic and position sense can lead to delays in the development of pencil grasp patterns in children with diplegic cerebral palsy [2].

Observations of the pencil grasp pattern at the age of six to seven years in diplegic CP patients reveal a significantly delayed palmer-supinated grasp compared to normal children in the same age group. Moreover, the 12-year-old child is between a static tripod and a dynamic tripod posture. Thus, the pencil grasp is not mature in the diplegic CP. Similarly, the drawing pattern also shows a significant difference between normal and diplegic CP. The CP children have shown a marked variation in the drawing pattern (t-value = 5.796; p-value = 0.001). At seven years of age, some diplegic CP children mark a drawing pattern of 2 to 2 1/2, whereas normal children achieve the same pattern at the appropriate age. The pattern of drawing is markedly dependent on the pencil grasp pattern. These studies have shown that if problem areas are identified and treated in the early age group as early intervention, it can improve sensory-motor functions in cerebral palsy children [13-15]. So, according to my study, CP children have inappropriate grasp and drawing pattern delays. For instance, a seven-year-old CP child has a grasp pattern of 1-1.5 years old, which is very delayed secondary to a lack of early intervention and sensory-motor delays. If a holistic approach is used and early intervention starts with team-based approaches, CP students can perform much better in their academics as well as gain independence in ADLs.

In another study, it was identified that functional difficulties are not only due to motor issues; both sensory and motor processing difficulties lead to poor writing and pre-writing skills [2,16]. Sensory information also plays an important role in prewriting and handwriting skills, which are also affected in CP children. In writing, movement of the hand and arm, hand positioning, hand skills, hand manipulation, pressure application, and feel of the pencil are also important aspects that affect CP children. The different conditions that may make prewriting skills difficult are: awkward pencil grasp; difficulty in controlling a pencil for drawing; tendency to use the whole hand to manipulate objects; poor endurance for pencil-based activities; difficulty in staying within the lines when coloring; inappropriate pressure on the paper for pencil-based activities; poor upper limb strength; difficulty in coordinating both hands for two-handed tasks; and poor hand-eye coordination.

The CP children demonstrate developmentally inappropriate patterns, i.e., palmer supinated grasp, at the age of seven years, whereas in normal children it is achieved at 1-1.5 years. In this study, it was found that impairments in sensory-motor aspects like proprioception, vestibular, bilateral coordination, speed dexterity of the upper extremity, visual and spatial perception, visual motor organization, and tactile sensory impairments affect the handwriting skills of children with cerebral palsy as compared to their peers [12]. According to our study, pencil grasp and drawing patterns are significantly delayed because of sensory-motor delays and a lack of intervention. For instance, diplegic CP in age groups six to seven is still at palmer-supinated grasp, which is significantly delayed when compared to normal children of the same age group in the study. Apart from that, CP children have scored lower on EDPA, which implies the need for therapies and early diagnosis with evaluation and treatment.

EDPA is a scale of hand function that evaluates the hand function, arm, and hand development of developmentally and physically disabled children. It assesses the hand function of children (age groups 0-6 years) with disabilities. An occupational therapist uses EDPA for treating and assessing hand functions in disabled children who are affected by sensory-motor and cognitive disabilities. Difficulty with hand functions affects the development of independence and the ability to learn in an academic and social environment [17,18].

Limitation

Our case is, however, limited by a few variables. The sample size was small for this study, and there was a large variation in the age of diplegic CP children. A larger group and sample size for a specific age can enhance the accuracy of the study and yield better results. This can be more accurate if the exact age group is selected for CP and normal children and compared with peers of the same age group. More standardized scales and testing can be included to increase the accuracy of the results.

## Conclusions

In conclusion, diplegic CP children performed lower on the EDPA and showed greater variation in the pencil grasp and drawing than the normal children. These are the important sensory-motor skills needed for prewriting and handwriting activities. It is affected in CP children because of neurological and physiological delayed development. If problem areas like range of motion, posture, grip, in-hand manipulation, strength, hand skills, etc., are assessed on time and early intervention is started at the initial development delay stage, CP children can perform much better in their academics as well as ADL (activities of daily living). This study emphasizes the importance of identifying the problem areas and starting early intervention.
